# Memory in language-impaired children with and without autism

**DOI:** 10.1186/s11689-015-9111-z

**Published:** 2015-06-14

**Authors:** Alison Presmanes Hill, Jan van Santen, Kyle Gorman, Beth Hoover Langhorst, Eric Fombonne

**Affiliations:** Center for Spoken Language Understanding, Oregon Health & Science University, Portland, OR USA; Institute on Development and Disability, Oregon Health & Science University, Portland, OR USA; Department of Pediatrics, Oregon Health & Science University, 3181 SW Sam Jackson Park Road, GH40, Portland, OR 97239 USA; Department of Behavioral Neuroscience, Oregon Health & Science University, Portland, OR USA; Department of Psychiatry, Oregon Health & Science University, Portland, OR USA

**Keywords:** Autism spectrum disorders, Specific language impairment, Verbal memory, Nonword repetition, Verbal working memory, Narrative, Processing speed

## Abstract

**Background:**

A subgroup of young children with autism spectrum disorders (ASD) have significant language impairments (phonology, grammar, vocabulary), although such impairments are not considered to be core symptoms of and are not unique to ASD. Children with specific language impairment (SLI) display similar impairments in language. Given evidence for phenotypic and possibly etiologic overlap between SLI and ASD, it has been suggested that language-impaired children with ASD (ASD + language impairment, ALI) may be characterized as having both ASD and SLI. However, the extent to which the language phenotypes in SLI and ALI can be viewed as similar or different depends in part upon the age of the individuals studied. The purpose of the current study is to examine differences in memory abilities, specifically those that are key “markers” of heritable SLI, among young school-age children with SLI, ALI, and ALN (ASD + language normal).

**Methods:**

In this cross-sectional study, three groups of children between ages 5 and 8 years participated: SLI (*n =* 18), ALI (*n =* 22), and ALN (*n =* 20). A battery of cognitive, language, and ASD assessments was administered as well as a nonword repetition (NWR) test and measures of verbal memory, visual memory, and processing speed.

**Results:**

NWR difficulties were more severe in SLI than in ALI, with the *largest* effect sizes in response to nonwords with the *shortest* syllable lengths. Among children with ASD, NWR difficulties were not associated with the presence of impairments in multiple ASD domains, as reported previously. Verbal memory difficulties were present in both SLI and ALI groups relative to children with ALN. Performance on measures related to verbal but not visual memory or processing speed were significantly associated with the relative degree of language impairment in children with ASD, supporting the role of verbal memory difficulties in language impairments among early school-age children with ASD.

**Conclusions:**

The primary difference between children with SLI and ALI was in NWR performance, particularly in repeating two- and three-syllable nonwords, suggesting that shared difficulties in early language learning found in previous studies do not necessarily reflect the same underlying mechanisms.

**Electronic supplementary material:**

The online version of this article (doi:10.1186/s11689-015-9111-z) contains supplementary material, which is available to authorized users.

## Background

Autism spectrum disorders (ASDs) are characterized by significant impairments in social reciprocity and communication, accompanied by patterns of repetitive, ritualistic behaviors or intense interests [1]. Although impairments in structural language abilities (phonology, grammar, vocabulary) may often be present in children with ASD, such impairments are not considered to be core symptoms of ASD [2]. Language impairment is also not specific to ASD [3]. In particular, children with specific language impairment (SLI) have significantly impaired language abilities in the absence of other developmental or sensory problems [3, 4]. Although clinical definitions clearly delineate SLI as distinct from ASD [5], much research has focused on the relationship (similarities and dissimilarities) between these two disorders [3, 6]. For example, family studies have shown that language-based difficulties are elevated in parents and siblings of individuals with ASD [7–9] and ASD risk is elevated in siblings of children with SLI [10]. Coupled with evidence that approximately 50 % of verbally fluent children with ASD also meet standard criteria for language impairment (henceforth, ASD with language impairment (ALI)) [11, 12], these findings suggest phenotypic as well as etiologic overlap between SLI and ASD [13–15], implying at least one or more shared genes [13].

The extent to which the language phenotypes in SLI and ALI can be viewed as similar or different, however, depends in part upon the age of the individuals studied [5]. Generally, the “typical” language phenotypes of preschool-aged children with ALI and SLI are thought to substantially overlap, whereas school-age children with ALI are thought to display more severe impairments in higher order processing as opposed to the profound difficulties in comprehension and production of language displayed by children with SLI [5]. Examining differences between children with SLI and ALI in the early school-age years may be particularly important because the linguistic and behavioral profiles for each disorder may shift over time from childhood into adolescence. For example, among a sample of individuals with autism and developmental language disorders followed longitudinally from early childhood to adulthood (ages 7–8 to 23–24, respectively), social and communication problems became more apparent in adulthood among those with language disorders, whereas language scores improved significantly more with age in the autism group than in the language-disordered group [16]. A more recent study with preschool-aged children (2–4 years) found that 76 % of those with ALI no longer met criteria for language impairment 12 months later [17]. Importantly, for children with ASD, language impairment measured at ages 6–8 years has been found to be a more robust predictor of symptom and functional outcomes than language measured at earlier ages [18], suggesting that language abilities during the early school-age years play a pivotal role in development. Given that the language phenotypes of children with SLI and ALI may be expected to overlap most significantly at younger ages and diverge over time, it is important to understand the nature of early language impairments in children with ASD and whether parallels in such impairments in children with SLI reflect shared mechanisms underlying difficulties in early language learning.

Recently, the study of clinical “markers” of SLI in children with ALI has further shed light on the potential overlap between the two disorders. One of the most robust findings is that children in both groups have difficulties with nonword repetition (NWR), which are interpreted to reflect underlying deficits in phonological memory [19, 20]. NWR ability is typically measured by tests that involve repeating syllable strings of nonwords (e.g., *tauveeb*) of increasing length presented aurally. Approximately 75 % of children with SLI have NWR difficulties [21], and such difficulties persist even after past language impairments have resolved [22]. Consequently, difficulties with NWR are considered to be a reliable behavioral marker of SLI, as well as a clinical marker of heritable language impairment [23]. Importantly, NWR performance in children with SLI degrades as syllable length increases: when nonwords are shorter (1 or 2 syllables), these children perform at levels commensurate to non-language-impaired children; when nonwords are longer (≥3 syllables), they perform significantly worse than unaffected children [20, 24].

Evidence that children with ALI also experience difficulties with NWR [15, 25–27] led some researchers to question whether a subgroup of children with ASD display the same neurocognitive phenotype that characterizes children with SLI [14]. On the other hand, others have argued that similarities between SLI and ALI have primarily been found on standardized assessments, which may not be sensitive to the ways that children with SLI and ALI differ, and do not necessarily reflect shared etiology [5, 27, 28]. In support of this view, recent studies have found evidence for different profiles of NWR difficulties as a function of syllable length in children with SLI and ALI. Specifically, children with ALI are better able to repeat nonwords with three or more syllables than those with SLI [26–28]. However, findings have been inconsistent across studies, likely due to methodological differences including specific features of the tests used to measure NWR (see Table 1). In addition, the samples in previous studies were either restricted to only those children with poor overall NWR performance [27] or to older children and adolescents [26, 28], limiting the generalizability of these results to younger children. Previous analyses of NWR as a function of syllable length have also not included a group of children with ALN, leaving some questions as to whether the patterns of NWR difficulties observed in ALI groups are linked to language impairment or whether the demands of NWR tasks may be particularly difficult for children with ASD regardless of language abilities [27].

**Table 1:** Characteristics of different tests of nonword repetition

	NEPSY	CTOPP	CNRep	NRT (current study)
	repetition of nonsense words			
Scoring method	• Scored online • Syllable-level: score is number of syllables repeated without errors • Errors defined as distortions or omissions of syllables (not insertions)	• Scored online • Word-level: score is number of whole words repeated without errors • Errors defined as phoneme distortions, omissions, or insertions	• Scored online • Word-level: score is number of whole words repeated without errors • Errors defined as phoneme distortions, omissions, or insertions	• Scored offline via transcripts • Phoneme-level: score is percentage of phonemes repeated without errors • Errors defined as distortions or omissions of phonemes (not insertions)
Total number of nonwords	13	30	40	12 (typically 16)
Range of syllable lengths	2–5	1–10	2–5	2–4 (typically 1 syllable words also included)
Articulatory complexity	High: most nonwords contain at least one consonant cluster (e.g., *crumsee*)	Mixed: 8 nonwords contain consonant clusters (e.g., *burloogugendaplo*)	Mixed: half of the nonwords contain consonant clusters (e.g., *prindle*)	Low: no consonant clusters, late-acquired phonemes not used
Wordlikeness	Mixed:	Low:	Mixed:	Low:
	• Some nonwords contain English words or affixes (e.g., *pledgyfriskree*, *inkewsment*) • Low phonotactic probabilities	• No embedded English words • Low phonotactic probabilities	• Some nonwords contain English words or affixes (e.g., *penl*, *glistering*) • High phonotactic probabilities	• No embedded English words • Low phonotactic probabilities
Stopping rule	Test is discontinued after 4 consecutive scores of 0.	Test is discontinued after 3 consecutive scores of 0.	None	None

*NEPSY* a developmental NEuroPSYchological assessment, *CTOPP* Comprehensive Test of Phonological Processing, *CNRep* Children’s Test of Nonword Repetition, *NRT* Nonword Repetition Test

Recently, Whitehouse and colleagues [27] hypothesized that NWR performance among children with ASD is uniquely impacted by the presence of significant impairments in multiple behavioral domains of ASD symptomatology. For example, the combination of difficulties attending to others’ speech and imitating others could result in poor NWR performance in children with ASD [27]. In their study, Whitehouse et al. [27] reported that nine out of nine children who performed poorly on a measure of NWR met their criteria for substantial impairments in two or three of the domains on the SCQ. However, this finding was based on a small sample and has yet to be replicated.

The degree to which neurocognitive phenotypes overlap in children with ALI and SLI beyond standardized language assessments and NWR is also unclear, particularly during the early school-age years. Numerous studies have now found that children with SLI have difficulties in verbal memory abilities such as in tests of simple digit span [22, 29–35], in addition to limitations in verbal working memory [19, 24, 29, 36–38] and processing speed [19, 22, 30, 39]. This constellation of impairments has led some researchers to hypothesize that language learning difficulties in children with SLI stem from limited processing abilities [19]. Importantly, memory limitations seem to be specific to the verbal domain [22] as visual memory is not significantly impaired in children with SLI [22, 40]. Given that children with ASD (regardless of language) have impaired verbal memory [41] and slower processing speed [42, 43], it is possible that the combination of language impairment and ASD constitutes a developmental “double hit” [11, 17], whereby children with ALI are more severely impacted in domains specifically related to memory and processing of verbal information relative to children with ALN or SLI.

### The current study

This study has three aims. Our primary aim is to determine whether young children with SLI (5–8 years) would display more severe NWR difficulties than same-aged children with ALI and whether the pattern of such difficulties across syllables would vary by group. We hypothesized that increasing nonword length in the NWR task would more negatively impact performance of children with SLI relative to the ALI and ALN groups, supporting the theory that SLI is associated with specific difficulty forming phonologically complex representations [28]. As a secondary aim, we examined Whitehouse and colleagues’ hypothesis [27, 44] that poor NWR performance in children with ASD is associated with the presence of significant impairments in behavioral domains associated with ASD, specifically, social affect and restricted, repetitive behaviors. Finally, we examined differences in broader memory abilities (verbal memory, verbal working memory, processing speed) between groups to further examine possible features of the neurocognitive phenotypes that may overlap between children with SLI and ALI.

## Methods

### Participants

Children aged 5–8 years from the Portland, OR area participated in this study. Participants were excluded if they had any of the following: (1) identified metabolic, neurological, or genetic disorder; (2) gross sensory or motor impairment; (3) brain lesion; or (4) orofacial abnormality (e.g., cleft palate). All participants spoke English as their native language, produced a mean length of utterance in morphemes (MLU-M) of at least three, and had performance IQ (PIQ) scores ≥80 (with the exception of one child in the SLI group who had a PIQ of 79 and core language score of 60 on the Clinical Evaluation of Language Fundamentals). IQ was measured using the Wechsler Preschool and Primary Scale of Intelligence (WPPSI-III) [45] for children between 5 and 6;11 years old, and the Wechsler Intelligence Scale for Children (WISC-IV) [46] for children age seven and older. During an initial screening procedure, a certified speech-language pathologist confirmed the absence of speech intelligibility impairments.

Children with SLI were recruited through local speech clinics, speech-language pathologists, and the Oregon Speech and Hearing Association. The following criteria were used to define SLI in this study: (1) documented history of language delay and/or deficits; (2) best estimate clinical (BEC) consensus judgment (two clinical psychologists, a speech-language pathologist, and an occupational therapist, all with specific expertise with ASD) of language impairment in the absence of ASD based on all available evidence, including medical and family history, our assessments, prior assessments, and education records; and (3) core language scores (CLS) on the Clinical Evaluation of Language Fundamentals (CELF) [47, 48] below 85 (1 SD below the mean). The CELF assesses morphology, syntax, semantics, and memory for language. For children under 6 years of age, the CELF Preschool-2 [47] was administered; the CELF 4 [48] was used for ages six and older. The subtests that contribute to the CLS have the greatest sensitivity for detecting language disorders in both the receptive language (RL) and expressive language (EL) domains. Subtests that contribute to the CLS in the CELF P-2 include sentence structure (RL), word structure (EL), and expressive vocabulary (EL), and in the CELF 4 CLS, they include concepts and following directions (RL), word structure (EL), recalling sentences (EL), and formulated sentences (EL).

Children with ASD were recruited through local healthcare specialists, education service districts, autism clinics, parent groups, and non-profit autism organizations. The following criteria were used to define ASD in this study: (1) an existing diagnosis of ASD; (2) a BEC judgment of ASD using the DSM-IV-TR criteria [49]; and (3) above cut-off scores for ASD on both the Autism Diagnostic Observation Schedule (ADOS-G Module 2 or 3) [50] revised algorithm [51] and the Social Communication Questionnaire (SCQ) [52] according to the recommended cut-off score of 12 for research purposes [53]. We subdivided the ASD group into those with language impairment (ALI) and those with normally developing language (ALN) using the same CELF CLS criterion as with SLI. These criteria led to three groups of children (see Table 2): SLI (*n =* 18), ALI (*n =* 22), and ALN (*n =* 20). Overall, 13 children received the CELF P-2 (SLI, *n =* 3; ALI, *n =* 5; ALN, *n =* 5) and 47 received the CELF 4 (SLI, *n =* 15; ALI, *n =* 17; ALN, *n =* 15). Table 2 shows that the SLI and ALI groups did not differ significantly from each other in terms of IQ (full-scale, PIQ, and VIQ) or language (PPVT-III, MLU-M, all CELF index scores), but did differ significantly as expected on measures specific to ASD symptoms (ADOS, SCQ, CCC-2 SIDI). We also note that approximately 90 % of children in each language-impaired group had scores on the CELF recalling sentences subtest more than one standard deviation below the normative mean (SLI, 16 out of 18; ALI, 20 out of 22), corresponding to scores below the 16th centile; scores in this range have been observed in approximately 82 % of children with SLI [33]. The ALI and ALN groups did differ on full-scale and verbal IQ (but not PIQ) and all language-related measures. There was also a significant difference on the CCC-2 SIDI.

**Table 2:** Participant characteristics

	Mean (SD)					
	SLI	ALI	ALN	*H*(2)	*p*	Post hoc adjusted
	*n* = 18 (11 male)	*n* = 22 (19 male)	*n* = 20 (18 male)			Mann–Whitney tests^a^
Age (years)	7.2 (0.8)	6.9 (1.0)	6.9 (1.1)	1.65	.44	
Full-scale IQ^b^	88.3 (7.8)	91.6 (9.0)	110.1 (13.7)	26.10	<.001	[SLI = ALI] < ALN
Performance IQ^b^	101.1 (12.3)	107.8 (13.6)	117.1 (14.5)	9.91	<.01	SLI < ALN^c^
Verbal IQ^b^	86.2 (6.1)	83.3 (10.3)	110.1 (13.8)	29.55	<.001	[SLI = ALI] < ALN
PPVT-III^b^	87.9 (10.0)	90.1 (14.9)	111.6 (16.2)	20.08	<.001	[SLI = ALI] < ALN
MLU-M	4.2 (0.9)	3.9 (0.8)	4.6 (1.1)	3.38	.19	
CELF core language score^b^	72.5 (9.0)	71.4 (12.9)	108.5 (13.7)	39.48	<.001	[SLI = ALI] < ALN
CELF receptive language index^b^	84.9 (10.3)	78.1 (13.6)	104.2 (15.8)	24.66	<.001	[SLI = ALI] < ALN
CELF expressive language index^b^	69.5 (9.9)	70.6 (12.4)	109.0 (13.1)	39.36	<.001	[SLI = ALI] < ALN
ADOS calibrated severity score^d^	3.1 (2.6)	7.8 (1.8)	6.8 (2.0)	24.16	<.001	SLI < [ALI = ALN]
Social Communication Questionnaire	12.1 (6.7)	21.1 (5.6)	21.0 (4.9)	17.52	<.001	SLI < [ALI = ALN]
VABS-II adaptive behavior composite^b^	87.0 (11.6)	81.9 (9.2)	81.5 (9.8)	2.89	.23	
CCC-2 general communication composite	47.3 (11.7)	46.4 (13.6)	53.0 (8.4)	3.38	.18	
CCC-2 Social Interaction Deviance Index (SIDI)^e^	6.3 (11.4)	−9.4 (7.2)	−15.1 (6.9)	27.25	<.001	SLI > ALI > ALN

*ADOS* autism diagnostic observation schedule, *CCC-2* Children’s Communication Checklist 2nd Edition, *CELF* Clinical Evaluation of Language Fundamentals (preschool 2nd Edition or IV), *PPVT-III* Peabody Picture Vocabulary Test 3rd Edition, *VABS-II* Vineland Adaptive Behavior Scales 2nd Edition

^a^Indicated pairwise group differences (< or >) based on *p* values adjusted using the False Discovery Rate method (*q* < .05) [63]

^b^Standard scores (*M* = 100, SD = 15)

^c^For PIQ, the ALI group did not differ significantly from either the SLI or the ALN groups

^d^This score ranges from 0–10 [59]

^e^Positive SIDI scores indicate primarily structural language difficulties; negative scores indicate primarily pragmatic/social difficulties

### Procedure

This research was approved by the Oregon Health & Science University Institutional Review Board. Participating families were fully informed about the study procedures and provided written consent. Participants completed a battery of experimental tasks and cognitive, language, and neuropsychological assessments over approximately six sessions of 2–3 h each.

### Measures

The development of the stimuli and procedures for the nonword repetition task have been described in detail elsewhere [54]. In the current study, children listened to pre-recorded nonwords (e.g., *tauveeb*) and were instructed to immediately repeat each item. Trials were administered in order of increasing syllable length. One-syllable nonwords were administered as practice trials and were not analyzed. Thus, NWR performance was assessed with a total of 12 trials, consisting of four nonwords at each of three syllable lengths (2, 3, 4). Children’s responses were audio recorded for offline phonetic transcription and scoring. Following Dollaghan and Campbell [54], a target phoneme was scored as incorrect if the child omitted that phoneme or substituted another English phoneme for it; any insertions were ignored, and subphonemic distortions were scored as correct. The number of phonemes repeated correctly across all trials for each syllable length was divided by the total number of target phonemes (2 syllables = 20 phonemes; 3 syllables = 28 phonemes; 4 syllables = 36 phonemes) to obtain the proportion of phonemes correct (PPC) for each syllable length. We also calculated a total PPC score, which is the total phonemes correct divided by the total number of target phonemes, to examine associations with ASD symptoms. All children in the SLI group received all four trials for each syllable length. Some children in the ALI group were missing data for one of the four trials (*n =* 2 for two-syllable-length trials; *n =* 1 for three-syllable-length trials; *n =* 1 for four-syllable-length trials); one child in the ALN group was missing one four-syllable trial. For these children, the PPC scores were calculated based on the three intact trials.

Additional verbal memory abilities were assessed using a digit span test (CELF Number Repetition Forward; *M* = 10, SD = 3), a backward digit span test (CELF Number Repetition Backward; *M* = 10, SD = 3), and a narrative memory test (NEPSY Narrative Memory [55]; *M* = 10, SD = 3). It is important to note that the CELF Number Repetition Forward and Backward tasks are only included in the CELF 4 (which is valid for children age 5 and over) but were administered to all children in the current study. Two visual memory subtests from the Wide-Ranging Assessment of Memory and Learning 2nd Edition (WRAML-2) [56] were also administered: Picture Memory and Finger Windows (both: *M* = 10, SD = 3). The Picture Memory subtest involves instructing children to look at a complex scene then look at a second, similar scene and identify what is now different. In the Finger Windows subtest, children watch as the examiner pokes a pencil eraser through a series of holes in a plastic card. For each trial, children are immediately asked to place a finger in each hole in the same order as the examiner, with the length of each sequence increasing on successive trials. Processing speed was assessed by scaled scores (*M* = 10, SD = 3) on two processing speed subtests from the WPPSI-III or the WISC-IV [45, 46]: Coding and Symbol Search. The Peabody Picture Vocabulary Test 3rd Edition (PPVT-III) [57] provided a measure of receptive vocabulary. The ADOS [50], a semi-structured autism diagnostic observation, was administered to all children in the current study and was scored according to the revised algorithms [51]. Calibrated severity scores (CSS) were calculated as an indicator of total ASD severity (total CSS; [58]) and severity of social affect (SA) and restricted and repetitive behavioral (RRB) symptoms [59]. The SA CSS reflects the severity of the child’s social communication impairments, whereas the RRB CSS reflects the severity of symptoms such as hand flapping, sensory examination of materials, or excessive references to a particular topic. MLU-M was based on transcriptions of child speech during the ADOS.

Parents completed the SCQ [52], the Vineland Adaptive Behavior Scales, 2nd Edition (VABS-II) [60], and the Children’s Communication Checklist (CCC-2) [61]. The SCQ is a 40-item questionnaire that assesses core autism symptoms. The VABS-II assesses functional skills used in everyday life and provides an adaptive behavior composite as an estimate of overall adaptive functioning (*M* = 100, SD = 15). The CCC-2 is a 70-item questionnaire that assesses both the structural and pragmatic aspects of a child’s communicative ability. The general communication composite (GCC) is the sum of eight subscales related to communication (speech, syntax, semantics, coherence, initiation, stereotyped language, use of context, and nonverbal communication). The Social Interaction Deviance Index (SIDI) reflects the relative extent of social communication versus structural language difficulties. Positive SIDI scores indicate primarily structural language difficulties; negative scores indicate primarily pragmatic/social difficulties.

### Statistical analyses

The goal of the analyses of NWR performance was to examine group differences as a function of syllable length. Because PPC scores for each syllable length were correlated, a multivariate analysis of covariance (MANCOVA) would have been the preferable analytic approach. Unfortunately, the distributions of PPC scores strongly deviated from the multivariate normal distribution, as did each syllable-length PPC score. Therefore, we first used the multivariate extension of the nonparametric Kruskal–Wallis test with PPC scores at each syllable length (2, 3, 4) as the dependent variables and the groups (SLI, ALI, ALN) as the between-subjects factor, implemented in the “MNM” package in R using *p* values estimated from 10,000 random permutations as recommended for small sample sizes [62]. Univariate Kruskal–Wallis tests (*H*) were then conducted at each individual syllable length followed by pairwise Mann–Whitney *U* tests to analyze group differences. Levene’s tests confirmed that the assumption of homogeneity of variances was met for PPC scores at all syllable lengths. Within each syllable length, a false discovery rate of *q* <.05 [63] was used to control for the planned contrasts (*n =* 3); all reported *p* values are adjusted using the FDR method.

To examine the effects of syllable length on NWR performance within each group, a series of planned Wilcoxon signed-rank tests were performed (adjusted *p* values are reported; *n* = 3 contrasts per group). For all between- and within-group contrasts, nonparametric effect sizes are reported as Cliff’s delta (Δ), implemented within the “orddom” package in R [64]. The value of this statistic reflects the degree of overlap between the two distributions of scores; a Δ of ±1.0 indicates the absence of overlap between the two groups, whereas a value of 0 indicates that the distributions of the two groups are identical. Although not directly comparable to Cohen’s *d* [65], Cliff’s Δ values of .147 correspond to a small effect size, .33 to a medium effect size, and .474 to a large effect size, had the two distributions been normally distributed [66].

Following Whitehouse et al. [27], in order to examine the hypothesis that poor NWR performance is associated with deficits in multiple ASD symptom domains, we classified children with ASD based on whether or not they had poor NWR performance following a median split of total PPC scores (≥ vs. <85 %; *n =* 19 and 23, respectively). We also classified the same children with ASD according to median splits of ADOS SA CSSs (7 or higher was categorized as high) and RRB CSSs (9 or 10 were categorized as high) [59]. We conducted a Fisher exact test on proportions (with both PPC and ADOS CSSs as categorical variables) and a Mann–Whitney *U* test on total PPC scores as a continuous dependent variable with the number of ASD domains impacted as measured by ADOS CSSs (zero or one vs. both categorized as “high” by median splits) as the independent factor. In Whitehouse et al. [27], a scaled score of 8 on the NEPSY NWR test was the criterion for poor performance on that test, which (assuming normally distributed scores) corresponded to approximately 1.6 SDs below the mean on this test from a previous study and .67 SDs below the mean based on the US scaled scores (*M* = 10, SD = 3). We used the median as the criterion in the current sample due to the non-normal, negatively skewed distribution of total PPC scores.

Across groups, age was not associated with PPC scores at any syllable length (Kendall’s τ range, .01–.07; *p*s > .80). PIQ was also not significantly associated with PPC scores for 2-syllable (τ = .14; *p* = .19), 3-syllable (τ = .21; *p* = .08), and 4-syllable nonwords (τ = .08; *p* = .41). Additional file 1 presents a follow-up analysis involving pairwise contrasts of NWR PPC scores with groups specifically matched on age (as the nonword repetition task does not provide norm-referenced scores) and PIQ according to a slightly more conservative criterion of Mann–Whitney *U* tests *p* ≥ .25.

For all other measures, age-normed scaled scores were available (*M* = 10, SD = 3). Prior to analysis, all variables were screened for normality and homogeneity of variances. Although the assumption of homogeneity of variances was met for all variables, several were not normally distributed. Therefore, we examined differences among the three groups using Kruskal–Wallis tests (*H*), followed by Mann–Whitney *U* tests to examine post hoc contrasts. Within each measure, *p* values are adjusted using the FDR method (*n* = 3 contrasts per variable).

In addition to categorical analyses, we were also interested in examining language as a continuous variable within the ASD group overall (*n* = 42) to determine whether performance on each measure accounted for significant variability in language abilities among children with ASD. Following Baird et al. [22], we calculated the difference between PIQ and CELF CLS scores (henceforth, the PIQ-CELF CLS discrepancy score), both of which have a mean of 100 (SD = 15), to estimate the relative degree of language difficulty in children with ASD (ALI and ALN combined): positive scores indicate higher PIQ than language scores; negative scores indicate higher language than PIQ scores. Kendall’s τ was used as the measure of association with PIQ-CELF CLS discrepancy scores.

## Results

### Nonword repetition: between-group differences

The multivariate Kruskal–Wallis test revealed a significant effect of group on NWR PPC scores, *H* = 23.01, *p* = .001. Univariate Kruskal–Wallis tests were significant at each syllable length (see Table 3). Separate pairwise Mann–Whitney *U* tests revealed that children with ALI had significantly higher PPC scores than those with SLI for both 2- and 3-syllable nonword stimuli with medium to large effect sizes (Δs ≥ 0.45; see Table 3). Children with ALN had significantly higher PPC scores than those with ALI for 3- and 4-syllable nonwords (Δs ≥ 0.46). Based on estimates for the modified probability of superiority effect size [67, 68], there was a 26.5 % chance that a participant randomly chosen from the SLI group would have a higher score than a randomly chosen child from the ALI group for 2-syllable nonwords, a 27.6 % chance for 3-syllable nonwords, and a 40 % chance for 4-syllable nonwords. As shown in Additional file 1, results (including magnitude of effect sizes) were similar when analyses were conducted on a subset of children in each group, pairwise group-matched to a stronger criterion of *p* ≥ .25 for age and PIQ. Additionally, we examined group differences according to a stricter criterion for language impairment defined by CELF CLS scores >1.25 SD below the norm (scores ≤81; *n* = 16 in each language-impaired group). Analyses revealed similar patterns of group differences and magnitude of effect sizes at 2 (*p* = .01, Δ = .54) and 3 syllables (*p* = .04, Δ = .43), but no differences for 4 syllable nonwords (*p* = .89, Δ = .03).

**Table 3:** Descriptive statistics and group contrasts for the proportion of phonemes correct on the nonword repetition test by syllable length

							Cliff’s Δ	
	SLI	ALI	ALN	*H*(2)	*p*	Post hoc adjusted	SLI < ALI	ALI < ALN
	(*n* = 18)	(*n* = 22)	(*n* = 20)			Mann–Whitney tests^a^		
2 syllables								
Median	.85	.95	.92	7.51	.02	SLI < [ALI = ALN]	.47	−.07
Range	.60–1.00	.80–1.00	.75–1.00					
Mean (SD)	.85 (.11)	.93 (.06)	.92 (.07)					
95 % CI for the mean^b^	[.80; .90]	[.91; .96]	[.89; .95]					
3 syllables								
Median	.79	.86	.96	18.43	<.001	SLI < ALI < ALN	.45	.46
Range	.50–.96	.57–1.00	.71–1.00					
Mean (SD)	.78 (.11)	.85 (.11)	.93 (.07)					
95 % CI for the mean^b^	[.72; .82]	[.80; .89]	[.89; .95]					
% PPC < .90^c^	89 %	73 %	35 %					
4 syllables								
Median	.60	.64	.81	14.46	<.001	[SLI = ALI] < ALN	.20	.48
Range	.33–.78	.36–.96	.53–.94					
Mean (SD)	.60 (.12)	.66 (.15)	.78 (.12)					
95 % CI for the mean^b^	[.54; .65]	[.59; .71]	[.72; .83]					
% PPC < .75^d^	89 %	73 %	40 %					

^a^Indicated pairwise group differences (< or >) based on *p* values adjusted using the False Discovery Rate method (*q* < .05) [63]

^b^Based on 10,000 bootstrapped samples

^c^Compared to 95 % of language-impaired children ages 5; 8 to 12; 2 (*N* = 44) based on enrollment in language intervention [54]

^d^Compared to 98 % of language-impaired children ages 5; 8 to 12; 2 (*N* = 44) based on enrollment in language intervention [54]

### Nonword repetition: within-group effect of syllable length

To better understand the effect of syllable length within each group, a series of paired Wilcoxon signed-rank tests were conducted between syllable lengths (*p* values adjusted using FDR for *n* = 3 contrasts; contrasts between 2- and 4-syllable nonwords were always significant and are not detailed further). For children in the SLI group, PPC scores decreased significantly with each increase in syllable length (3 syllables < 2: Δ = .67, *p* < .001; 4 syllables < 3: Δ = .78, *p* < .001). A similar pattern was true for children in the ALI group (3 syllables < 2: Δ = .59, *p* = .003; 4 syllables < 3: Δ = .82, *p* < .001). For those in the ALN group, PPC scores significantly decreased from 3- to 4-syllable nonwords (Δ = .90, *p* < .001) but not from 2- to 3-syllable nonwords (Δ = .15, *p* = .48). For contrasts between 2- and 3-syllable nonwords, the between-syllable effect size was greatest in the SLI group, although the between-syllable effect sizes in comparisons of 3- versus 4-syllable nonwords were comparably large in magnitude across all three groups (Δs ≥ .78), with 4-syllable nonwords consistently eliciting the lowest NWR performance levels.

### Nonword repetition and ASD symptom severity

Overall, 13 children with ASD received high scores on both domain CSSs (SA and RRB), 21 on either, and 8 on neither; participants with one or neither CSS categorized as high were combined for analyses due to low cell frequencies (also see [27]). A 2 × 2 (low/high PPC scores × 0-1/2 CSS domains high) Fisher exact test was not significant (*p* = .32). A Mann–Whitney *U* test with PPC scores as a continuous dependent variable revealed no differences in PPC scores between children with ASD who had either zero or one versus both ADOS CSSs categorized as high (*p* = .13; Δ = .30).

### Memory abilities and language impairment

Table 4 presents results of between-group analyses for measures related to verbal memory, visual memory, and processing speed. Children with ALI had significantly lower scores relative to those with ALN on the three measures related to verbal memory (CELF Number Repetition Forward, NEPSY Narrative Memory, and CELF Number Repetition Backward; Δs > .60), but did not differ from children with ALN on tests related to visual memory or processing speed. The only significant difference between children with SLI and ALI was on the NEPSY Narrative Memory test, where children with ALI received significantly lower scores than those with SLI (Δ = −.39). Results were relatively unchanged when more stringent criteria for language impairment were used (CELF CLS < 1.25 SD below the normative mean): only scores on narrative memory were lower for those with ALI than SLI (Δ = −.58).

**Table 4:** Means (standard deviations), group comparisons, and associations with degree of language impairment in ASD

	Median						Cliff’s Δ		ASD PIQ-CELF CLS discrepancy *τ* ^b^
	Mean (SD)								
Assessment	SLI	ALI	ALN	*H*(2)	*p*	Post hoc adjusted	SLI < ALI	ALI < ALN	
	*n* = 18	*n* = 22	*n* = 20			Mann–Whitney tests^a^			
CELF Number Repetition Forward^c^	7.0	8.0	10.0	19.36	<.001	[SLI = ALI] < ALN	.21	.65	−.53**
	6.83 (1.95)	7.36 (2.26)	10.50 (3.14)						
NEPSY Narrative Memory^c^	6.0	3.5	10.0	22.77	<.001	ALI < SLI < ALN	−.39	.77	−.53**
	5.89 (2.59)	4.05 (2.98)	9.40 (3.19)						
CELF Number Repetition Backward^c^	7.5	7.5	12.0	16.54	<.001	[SLI = ALI] < ALN	.04	.61	−.42*
	7.33 (3.18)	7.64 (3.54)	11.55 (2.93)						
Coding^c^	8.0	8.0	7.5	0.01	.99				.00
	8.28 (3.75)	8.50 (3.43)	8.25 (3.02)						
Symbol search^c^	7.5	8.0	8.0	1.99	.37				−.01
	6.83 (3.24)	8.09 (2.37)	8.05 (2.67)						
WRAML-II Picture Memory^c^	11.5	9.5	8.0	5.31	.07				.09
	10.56 (3.48)	9.14 (4.95)	7.75 (3.24)						
WRAML-II Finger Windows^c^	11.0	10.0	11.0	1.83	.40				.18
	9.89 (2.65)	9.77 (2.58)	10.95 (3.32)						

*CELF* Clinical Evaluation of Language Fundamentals, *NEPSY* a developmental NEuroPSYchological assessment, *WRAML-II* Wide-Range Assessment of Memory and Learning 2nd Edition

* *p* < .05; ** *p* ≤ .001

^a^Indicated pairwise group differences (< or >) based on *p* values adjusted using the False Discovery Rate method (*q* < .05) [63]

^b^For both ASD groups combined (ALI + ALN), Kendall’s tau between the degree of language impairment (measured by PIQ-CELF Core Language Score discrepancy score) and the assessment score is reported: negative values indicate that more severe relative language impairments are associated with poorer performance on that assessment

^c^Scaled scores (*M* = 10, SD = 3)

For children with ASD (ALI and ALN combined), the degree of language impairment (measured using the PIQ-CELF CLS discrepancy scores) was significantly associated with poorer performance on only the three verbal memory measures (*τ*s ≤ −.42); it was not associated with measures of processing speed or visual memory.

## Discussion

This study presents the first comparisons of language-related memory abilities among early school-age children (5–8 years) with SLI, ALI, and ALN. Our primary aim was to examine differences in patterns of NWR abilities between the three groups of children. Using the nonword repetition task [54], children with SLI produced significantly more phoneme errors compared to those with ALI in response to 2- and 3-syllable nonwords. These findings are consistent with recent research demonstrating greater impairments in nonword repetition abilities among children with SLI versus ALI [26–28], and suggests that, despite parallel impairments in core language as measured by the CELF standardized assessment, the cognitive or linguistic underpinnings of early language difficulties during the early school-age years may be distinct in children with SLI and ALI.

An alternative explanation is that, because we did not include a standardized measure of articulation, children with SLI had more difficulty articulating the nonwords than the ASD groups, as articulation difficulties are known to be more common among children with SLI than ALI (see [69]). However, greater articulation difficulties in children with SLI are unlikely to fully account for the group differences found in the current study for several reasons. First, a certified speech-language pathologist conducted an initial screening to rule out articulation and other oro-motor difficulties. Second, following the scoring guidelines [54], phoneme distortions explicitly did not affect PPC scores.

On the other hand, our results were not consistent with previous studies demonstrating that differences in nonword repetition between children with SLI versus ALI are greatest at longer syllable lengths [26–28]. Rather, we found the reverse: the effect size for the comparison between SLI and ALI was the greatest for 2-syllable nonwords, whereas the greatest difference in NWR performance between ALN and ALI was for 4-syllable nonwords. There are several possible explanations for the relatively poorer performance of children with SLI at the *shortest* syllable length. One possibility is that previous tasks may have included high wordlikeness items (Table 1), which could have supported performance among children with SLI in particular at shorter syllable lengths, masking NWR difficulties that may be apparent at longer syllable lengths [21]. Another possibility is that previous studies have tended to rely on measures that utilize word-level rather than phoneme-level scoring (Table 1), which could dilute subtle group differences in NWR performance that may be present at shorter syllable lengths [26]. Age also likely plays an important role here. We know very little about how NWR abilities develop in childhood and into adolescence in either language-impaired or typical children [21]. It is possible that we would observe different developmental trajectories of NWR abilities with age. For example, children with SLI may develop strategies and skills for repeating shorter nonwords yet continue to struggle to repeat longer nonwords into adolescence, while early language impairments (alongside NWR difficulties) in children with ALI may ameliorate to some extent during the same time period [5].

We also observed striking similarities between the three groups in terms of the effect of syllable length on NWR performance. While children with SLI showed a larger decrement in NWR performance in response to 2- versus 3-syllable nonwords relative to the two ASD groups, all three groups showed significant decrements in NWR performance in response to 3- versus 4-syllable nonwords with large effect sizes (Δs ≈ .8). This finding contrasts with previous research demonstrating greater effects of syllable length in children with SLI versus ALI [26, 27]. That is, in previous studies, children with SLI were relatively more affected by longer syllable lengths. However, Williams et al. [28] recently found that syllable length appeared to affect NWR performance in individuals with SLI (mean age = 12.4 years) somewhat less than in the other groups, including an ALI group. Given the younger age and more narrow age range of our sample, it is possible that repeating nonword stimuli with four or more syllables may be a developmentally difficult task; in the ALN group, for example, the maximum PPC scores for 2- and 3-syllable nonwords were higher than 95 %, whereas the highest PPC score for 4-syllable nonwords dropped to 84 %. Our results indicate that children in all three groups were equally affected by the increase from 3 to 4 syllables in the sense that all exhibited substantial decreases in their ability to repeat nonword phonemes accurately. Studies including participants without neurodevelopmental disorders would help to clarify developmental expectations regarding NWR performance as a function of syllable length.

As a secondary aim, we examined the hypothesis [27, 44] that NWR difficulties among children with ASD stem from the presence of significant impairments in multiple behavioral domains of ASD symptomatology. Our results did not support this hypothesis, but instead suggested that the severity of clinician-observed ASD symptoms is not strongly associated with the severity of NWR difficulties. This is in contrast to previous results reported by Whitehouse and colleagues [27]. Several factors may account for these dissonant findings. First, our sample age range is younger and narrower than that of Whitehouse and colleagues (6–15 years; mean age ≈ 10 years). Given possible improvements in language impairments among children with ALI into adolescence [5], it may be that the older group of children with autism and language impairment in their study included those with more persistent and severe language impairments than in the current sample. Alternatively, parents of children with ASD may perceive and report elevated symptoms on the SCQ in the presence of language impairment, whereas clinicians may be better able to evaluate ASD symptoms independent of language status. However, we did not find evidence in our sample for mean-level differences on either parent-reported (SCQ) or clinician-observed (ADOS) measures of ASD symptoms between ALI and ALN. No difference in NWR performance was found between children with ALN and ALI for 2-syllable nonwords, with both groups repeating over 92 % of phonemes correctly. This suggests that the NWR task itself was not difficult to understand for children with ASD. Poor NWR performance, however, can arise for a number of reasons including difficulties with speech processing, motor planning, and lexical and phonological knowledge [21, 70]. For children with ASD, it is possible that additional factors unmeasured in the current study, such as attention, impulsivity, and abilities related to executive control such as planning or inhibition may be found to influence NWR performance.

We also examined possible features of broader memory abilities (verbal memory, verbal working memory, processing speed) that may overlap between children with SLI and ALI. Consistent with several previous studies of children with SLI [22, 29–35], we found that this group had significantly lower scores on forward and backward digit span tests compared to the ALN group. Children with ALI also showed similar difficulties on each of these tests. This suggests a possible mechanism for language impairment in children with ASD related to restriction in working memory capacity that may be specific to verbal information. Both the categorical analysis and the associations between degree of language impairment (CELF CLS-PIQ discrepancy scores) converge in showing that verbal memory-related difficulties (forward/backward digit span, narrative memory) are uniquely associated with language impairment in children with ASD. However, the direction of influence is still unknown; for example, language learning difficulties could stem from memory and processing limitations, or limited language knowledge could hinder the growth of memory and processing capacities [38]. No differences were found on subtests related to processing speed, which is thought to be a domain of difficulty for children with SLI [19, 22, 30, 39]. However, because we did not include a group of typically developing children, it is difficult to draw inferences about these subtests as all group means were lower than the normative mean yet not by more than one standard deviation. Processing speed may be an area of weakness for children with neurodevelopmental disorders that is not specific to those with language impairment. Narrative memory, on the other hand, was a specific area of difficulty for children with ALI compared to both the SLI and ALN groups, providing support for the dual roles of structural and pragmatic language abilities in story comprehension and recall [71]. In fact, 20 % of children in the ALN group received scores lower than 1 SD on narrative memory, compared to 82 % of the ALI group.

However, as Tomblin points out [3], one question that remains unresolved is why there are so many children with ASD who may be considered “poor language learners.” In the current study, defining a separate group of children with ALI by applying the same criterion as for SLI resulted in 52 % of the ASD participants classified as language impaired, consistent with prevalence estimates of approximately 50 % based on epidemiological samples [11, 12]. Children with ASD may be particularly prone to language impairment, but for qualitatively different reasons than in the general population. For example (as one reviewer suggested), early experience listening and attending to speech is likely a critical ingredient for successful early language learning. In some children with ASD, reduced social engagement and attention to speech, combined with verbal memory and working memory difficulties, could result in language learning difficulties, leading to impairments in language that resemble those seen in children with SLI. However, although children in the ALN group did not have impaired language based on CELF Core Language Scores, it may not be accurate to characterize their language development as typical or unimpaired.

Strengths of this study include our focus on a relatively narrower age range compared to previous studies, which may have reduced the role of age as a confounding factor. We also included an array of verbal memory measures in addition to nonword repetition including digit span, backward digit span, and narrative memory, as well as measures related to memory for visual information and processing speed. Finally, our analyses also included children with ASD without language impairment (ALN), a group that has rarely been included in previous studies as a comparison for an ALI group. This allowed us to examine whether performance on NWR and other memory tasks among children with ALI was associated with the presence and severity of language impairment. These analyses showed that language impairment in children with ASD was systematically associated with poorer verbal memory and verbal working memory abilities.

This study also had several limitations. First, this was a cross-sectional study with an age range (5–8 years) that encompassed children at different stages of language development and as a result may have obscured important group and individual differences. This may have been exacerbated by our use of two different versions of the CELF [47, 48] to identify language impairment. Second, our sample sizes were small, and although not unusual in studies that involve comprehensive assessments, this may limit the generalizability of our findings. A related limitation is that our sample was predominantly male and we lacked statistical power and access to demographic data to examine the effects of gender, socio-economic status, race, or ethnicity, although these factors may influence many of the domains measured. Third, participants had IQ scores in the normal range, and therefore are not representative of the general ASD population. Language impairment in these individuals may differ qualitatively from language impairment in individuals with poorer levels of cognitive functioning [72]. Fourth, we used a “cut-off” approach as one of the three criteria for defining SLI in this study and to classify language impairment in children with ASD, following several previous studies [25, 73, 74] in using the criterion of 1 SD below the normative mean. Other studies have implemented more stringent criterion levels. In addition, some have incorporated NWR difficulties as one of the criteria for defining language impairment in SLI and ALI [15, 25, 27]. Our results suggest that NWR difficulties may have a different meaning in children with and without ASD, and may result in non-comparable definitions or inclusion criteria that in turn might bias the results. Finally, we did not include spontaneous language measures [75–78] or story generation abilities [79], all of which have substantial clinical utility in language-disordered populations and may further reveal meaningful differences between children with SLI, ALI, and ALN.

## Conclusions

This study adds to the literature by demonstrating both shared and diverging features of the neurocognitive phenotypes in young children with SLI and ALI. Language impairment, regardless of ASD diagnosis, was associated with difficulties in verbal memory and verbal working memory, whereas visual memory and processing speed were not robustly associated with the presence or severity of language impairment in ASD. The primary difference between children with SLI and ALI was in NWR performance, particularly in repeating 2- and 3-syllable nonwords, suggesting that shared difficulties in early language learning found in previous studies do not necessarily reflect the same underlying mechanisms. However, we do not know if these NWR difficulties persist over time in children with ALI, what factors may be associated with gains in NWR abilities, and whether such gains in turn impact language. Longitudinal studies are needed to better understand how the developmental language profiles change over time in both language-impaired and ASD populations.

## Additional file

Additional file 1:
**Matched group (age and PIQ) contrasts for the proportion of phonemes correct on the nonword repetition test by syllable length.**

## Abbreviations

ADOS: Autism Diagnostic Observation Schedule
ALI: ASD plus language impairment
ALN: ASD with normal language
ASD: autism spectrum disorder
CCC-2: Children’s Communication Checklist 2nd Edition
CELF: Clinical Evaluation of Language Fundamentals
NEPSY: a developmental NEuroPSYchological assessment
NWR: nonword repetition
PIQ: performance intelligence quotient
PM: phonological memory
PPVT-III: Peabody Picture Vocabulary Test 3rd Edition
SIDI: CCC-2 Social Interaction Deviance Index
SLI: specific language impairment
SNRep: sentence repetition
VABS-II: Vineland Adaptive Behavior Scales 2nd Edition
VIQ: verbal intelligence quotient
WM: working memory
WRAML-II: Wide-range Assessment of Memory and Learning 2nd Edition

## References

[1] American Psychiatric Association (2013). Diagnostic and Statistical Manual of Mental Disorders.

[2] Lord C, Jones RM (2012). Annual research review: re-thinking the classification of autism spectrum disorders. J Child Psychol Psychiatry.

[3] Tomblin B (2011). Co-morbidity of autism and SLI: kinds, kin and complexity. Int J Lang Commun Disord.

[4] Leonard LB (2000). Children with specific language impairment.

[5] Williams D, Botting N, Boucher J (2008). Language in autism and specific language impairment: Where are the links?. Psychol Bull.

[6] Bishop DVM (2010). Overlaps between autism and language impairment: phenomimicry or shared etiology?. Behav Genet.

[7] Fombonne É, Bolton P, Prior J, Jordan H, Rutter M (1997). A family study of autism: cognitive patterns and levels in parents and siblings. J Child Psychol Psychiatry.

[8] Piven J, Palmer P, Jacobi D, Childress D, Arndt S (1997). Broader autism phenotype: evidence from a family history study of multiple-incidence autism families. Am J Psychiatry.

[9] Schmidt GL, Kimel LK, Winterrowd E, Pennington BF, Hepburn SL, Rojas DC (2008). Impairments in phonological processing and nonverbal intellectual function in parents of children with autism. J Clin Exp Neuropsychol.

[10] Tomblin JB, Hafeman LL, O’Brien M (2003). Autism and autism risk in siblings of children with specific language impairment. Int J Lang Commun Disord.

[11] Loucas T, Charman T, Pickles A, Simonoff E, Chandler S, Meldrum D (2008). Autistic symptomatology and language ability in autism spectrum disorder and specific language impairment. J Child Psychol Psychiatry.

[12] Leyfer OT, Tager-Flusberg H, Dowd M, Tomblin JB, Folstein SE (2008). Overlap between autism and specific language impairment: comparison of autism diagnostic interview and autism diagnostic observation schedule scores. Autism Res.

[13] Groen WB, Zwiers MP, van der Gaag R-J, Buitelaar JK (2008). The phenotype and neural correlates of language in autism: an integrative review. Neurosci Biobehav Rev.

[14] Tager-Flusberg H, Joseph RM (2003). Identifying neurocognitive phenotypes in autism. Philosophical Transactions Royal Soc London.

[15] Kjelgaard MM, Tager-Flusberg H (2001). An investigation of language impairment in autism: implications for genetic subgroups. Lang Cogn Process.

[16] Mawhood L, Howlin P, Rutter M (2000). Autism and developmental receptive language disorder—a comparative follow-up in early adult life I: cognitive and language outcomes. J Child Psychol Psychiatry.

[17] Bennett TA, Szatmari P, Georgiades K, Hanna S, Janus M, Georgiades S (2014). Language impairment and early social competence in preschoolers with autism spectrum disorders: a comparison of DSM-5 profiles. J Autism Dev Disord.

[18] Bennett T, Szatmari P, Bryson S, Volden J, Zwaigenbaum L, Vaccarella L (2008). Differentiating autism and Asperger syndrome on the basis of language delay or impairment. J Autism Dev Disord.

[19] Leonard LB, Ellis Weismer S, Miller CA, Francis DJ, Tomblin JB, Kail RV (2007). Speed of processing, working memory, and language impairment in children. J Speech Lang Hear Res.

[20] Gathercole SE, Baddeley AD (1990). Phonological memory deficits in language disordered children: is there a causal connection?. J Mem Lang.

[21] Graf-Estes K, Evans JL, Else-Quest NM (2007). Differences in the nonword repetition performance of children with and without specific language impairment: a meta-analysis. J Speech Lang Hear Res.

[22] Baird G, Dworzynski K, Slonims V, Simonoff E (2010). Memory impairment in children with language impairment. Dev Med Child Neurol.

[23] Bishop DVM, North T, Donlan C (1996). Nonword repetition as a behavioural marker for inherited language impairment: evidence from a twin study. J Child Psychol Psychiatry.

[24] Montgomery JW (2000). Verbal working memory and sentence comprehension in children with specific language impairment. J Speech Lang Hear Res.

[25] Lindgren KA, Folstein SE, Tomblin JB, Tager-Flusberg H (2009). Language and reading abilities of children with autism spectrum disorders and specific language impairment and their first-degree relatives. Autism Res.

[26] Riches NG, Loucas T, Baird G, Charman T, Simonoff E (2011). Non-word repetition in adolescents with specific language impairment and autism plus language impairments: a qualitative analysis. J Commun Disord.

[27] Whitehouse AJO, Barry JG, Bishop DVM (2008). Further defining the language impairment of autism: is there a specific language impairment subtype?. J Commun Disord.

[28] Williams D, Payne H, Marshall C (2013). Non-word repetition impairment in autism and specific language impairment: evidence for distinct underlying cognitive causes. J Autism Dev Disord.

[29] Archibald LMD, Gathercole SE (2006). Short-term and working memory in specific language impairment. Int J Lang Commun Disord.

[30] Montgomery JW, Windsor J (2007). Examining the language performances of children with and without specific language impairment: contributions of phonological short-term memory and speed of processing. J Speech Lang Hear Res.

[31] Ellis Weismer S, Tomblin JB, Zhang X, Buckwalter P, Chynoweth JG, Jones M (2000). Nonword repetition performance in school-age children with and without language impairment. J Speech Lang Hear Res.

[32] Conti-Ramsden G (2003). Processing and linguistic markers in young children with specific language impairment (SLI). J Speech Lang Hear Res.

[33] Ramsden GC, Botting N (2001). Psycholinguistic markers for specific language impairment (SLI). J Child Psychol Psychiatry.

[34] Riches NG, Loucas T, Baird G, Charman T, Simonoff E (2010). Sentence repetition in adolescents with specific language impairments and autism: an investigation of complex syntax. Int J Lang Commun Disord.

[35] Riches NG (2012). Sentence repetition in children with specific language impairment: an investigation of underlying mechanisms. Int J Lang Commun Disord.

[36] Hoffman LM, Gillam RB (2004). Verbal and spatial information processing constraints in children with specific language impairment. J Speech Lang Hear Res.

[37] Marton K, Schwartz RG (2003). Working memory capacity and language processes in children with specific language impairment. J Speech Lang Hear Res.

[38] Ellis Weismer S, Evans J, Hesketh LJ (1999). An examination of verbal working memory capacity in children with specific language impairment. J Speech Lang Hear Res.

[39] Miller CA, Kail R, Leonard LB, Tomblin JB (2001). Speed of processing in children with specific language impairment. J Speech Lang Hear Res.

[40] Lum JAG, Conti-Ramsden G, Page D, Ullman MT (2012). Working, declarative and procedural memory in specific language impairment. Cortex.

[41] Bennetto L, Pennington BF, Rogers SJ (1996). Intact and impaired memory functions in autism. Child Dev.

[42] Mayes SD, Calhoun SL (2007). Learning, attention, writing, and processing speed in typical children and children with ADHD, autism, anxiety, depression, and oppositional-defiant disorder. Child Neuropsychol.

[43] Oliveras-Rentas RE, Kenworthy L, Roberson RB, Martin A, Wallace GL (2012). WISC-IV profile in high-functioning autism spectrum disorders: impaired processing speed is associated with increased autism communication symptoms and decreased adaptive communication abilities. J Autism Dev Disord.

[44] Whitehouse AJO, Barry JG, Bishop DVM (2007). The broader language phenotype of autism: a comparison with specific language impairment. J Child Psychol Psychiatry.

[45] Wechsler D (2002). Wechsler Preschool and Primary Scale of Intelligence.

[46] Wechsler D (2003). Wechsler Intelligence Scale for Children.

[47] Semel EM, Wiig EH, Secord W (2004). Clinical Evaluation of Language Fundamentals-Preschool-2. Second.

[48] Semel EM, Wiig EH, Secord W (2003). Clinical Evaluation of Language Fundamentals-Fourth Edition. Fourth.

[49] American Psychiatric Association (2000). Diagnostic and Statistical Manual of Mental Disorders.

[50] Lord CC, Risi SS, Lambrecht LL, Cook EHE, Leventhal BLB, DiLavore PCP (2000). The autism diagnostic observation schedule-generic: a standard measure of social and communication deficits associated with the spectrum of autism. J Autism Dev Disord.

[51] Gotham K, Risi S, Pickles A, Lord C (2007). The autism diagnostic observation schedule: revised algorithms for improved diagnostic validity. J Autism Dev Disord.

[52] Rutter M, Bailey A, Lord C (2003). The Social Communication Questionnaire: manual.

[53] Lee L-C, David AB, Rusyniak J, Landa R, Newschaffer CJ (2007). Performance of the social communication questionnaire in children receiving preschool special education services. Res Autism Spectrum Disorders.

[54] Dollaghan C, Campbell TF (1998). Nonword repetition and child language impairment. J Speech Lang Hear Res.

[55] Korkman M, Kirk U, Kemp S (1998). NEPSY: a developmental neuropsychological assessment.

[56] Sheslow D, Adams W (2003). Wide range assessment of memory and learning (WRAML2).

[57] Dunn LM, Dunn LM (1997). Peabody Picture Vocabulary Test-Third Edition.

[58] Gotham K, Pickles A, Lord C (2009). Standardizing ADOS scores for a measure of severity in autism spectrum disorders. J Autism Dev Disord.

[59] Hus V, Gotham K, Lord C (2012). Standardizing ADOS domain scores: separating severity of social affect and restricted and repetitive behaviors. J Autism Dev Disord.

[60] Sparrow SS, Balla DA, Cicchetti DV (2005). Vineland Adaptive Behavior Scales.

[61] Bishop DVM (2003). The Children’s Communication Checklist (CCC-2).

[62] Nordhausen K, Oja H (2011). Multivariate L1 methods: the package MNM. J Stat Software.

[63] Benjamini Y, Hochberg Y (1995). Controlling the false discovery rate: a practical and powerful approach to multiple testing. J Royal Stat Soc Series B (Methodological).

[64] Rogmann JJ: Ordinal Dominance Statistics (orddom): An R Project for Statistical Computing package to compute ordinal, nonparametric alternatives to mean comparison (Version 3.1). http://artax.karlin.mff.cuni.cz/r-help/library/orddom/html/orddom-package.html.

[65] Cohen J (1988). Statistical Power Analysis for the Behavioral Sciences.

[66] Grissom RJ, Kim JJ (2005). Effect sizes for research.

[67] Vargha A, Delaney HD (1998). The Kruskal-Wallis test and stochastic homogeneity. J Educ Behav Stat.

[68] Delaney HD, Vargha A (2002). Comparing several robust tests of stochastic equality with ordinally scaled variables and small to moderate sized samples. Psychol Methods.

[69] Williams D, Happé F, Jarrold C (2008). Intact inner speech use in autism spectrum disorder: evidence from a short-term memory task. J Child Psychol Psychiatry.

[70] Coady JA, Evans JL (2008). Uses and interpretations of non-word repetition tasks in children with and without specific language impairments (SLI). Int J Lang Commun Disord.

[71] Suh J, Eigsti I-M, Naigles L, Barton M, Kelley E, Fein D (2014). Narrative performance of optimal outcome children and adolescents with a history of an autism spectrum disorder (ASD). J Autism Dev Disord.

[72] Boucher J, Mayes A, Bigham S, Boucher J, Bowler D (2008). Memory, language and intellectual ability in low-functioning autism. Memory in Autism.

[73] Conti-Ramsden G, Simkin Z, Botting N (2006). The prevalence of autistic spectrum disorders in adolescents with a history of specific language impairment (SLI). J Child Psychol Psychiatry.

[74] Taylor LJ, Maybery MT, Grayndler L, Whitehouse AJO (2013). Evidence for distinct cognitive profiles in autism spectrum disorders and specific language impairment. J Autism Dev Disord.

[75] Condouris K, Meyer E, Tager-Flusberg H (2003). The relationship between standardized measures of language and measures of spontaneous speech in children with autism. Am J Speech Lang Pathol.

[76] Tager-Flusberg H, Rogers S, Cooper J, Landa R, Lord C, Paul R (2009). Defining spoken language benchmarks and selecting measures of expressive language development for young children with autism spectrum disorders. J Speech Lang Hear Res.

[77] Roberts JA, Rice ML, Tager-Flusberg H (2004). Tense marking in children with autism. Applied Psycholinguistics.

[78] Eigsti I-M, Bennetto L, Dadlani MB (2007). Beyond pragmatics: morphosyntactic development in autism. J Autism Dev Disord.

[79] Norbury CF, Bishop DVM (2003). Narrative skills of children with communication impairments. Int J Lang Commun Disord.

