# Supplementary material for: Memory in language-impaired children with and without autism
Source: J Neurodev Disord. 2015 Jun 14;7(1):19. doi: 10.1186/s11689-015-9111-z (PMC4472418; doi:10.1186/s11689-015-9111-z)
Supplement: Additional file 1: — Matched group (age and PIQ) contrasts for the proportion of phonemes correct on the nonword repetition test by syllable length. [file 11689_2015_9111_MOESM1_ESM.docx]

Appendix 1

Matched group (age and PIQ) contrasts for the proportion of phonemes correct on the nonword repetition test by syllable length

|  | Matched Groups^a^ | | Mann-Whitney *Z* statistic | Cliff’s Δ | Matched Groups^b^ | | Mann-Whitney *Z* statistic | Cliff’s Δ |
| --- | --- | --- | --- | --- | --- | --- | --- | --- |
|  | SLI  (*n* = 17) | ALI  (*n* = 21) |  |  | ALI  (*n* = 20) | ALN  (*n* = 18) |  |  |
| 2 syllables |  |  |  |  |  |  |  |  |
| *Median* | .85 | .95 | 2.54* | .48 | .95 | .95 | 0.54 | -.10 |
| *Range* | .60-1.00 | .80-1.00 |  |  | .80-1.00 | .75-1.00 |  |  |
| *Mean* (*SD*) | .85 (.11) | .94 (.06) |  |  | .94 (.06) | .92 (.07) |  |  |
| 3 syllables |  |  |  |  |  |  |  |  |
| *Median* | .79 | .86 | 2.36* | .45 | .86 | .96 | -2.45* | .46 |
| *Range* | .50-.96 | .57-1.00 |  |  | .61-1.00 | .71-1.00 |  |  |
| *Mean* (*SD*) | .78 (.12) | .86 (.11) |  |  | .86 (.09) | .93 (.08) |  |  |
| 4 syllables |  |  |  |  |  |  |  |  |
| *Median* | .61 | .64 | 1.06 | .20 | .62 | .78 | -2.51* | .48 |
| *Range* | .33-.78 | .36-.96 |  |  | .36-.96 | .53-.94 |  |  |
| *Mean* (*SD*) | .60 (.12) | .66 (.15) |  |  | .64 (.15) | .77 (.13) |  |  |

** p*-value < .05 adjusted using the False Discovery Rate method (*q* < .05) {Benjamini:1995kt}.

^a^ Age (*p* = .51) and PIQ (*p* = .42) did not differ between SLI and ALI groups.

^a^ Age (*p* = .93) and PIQ (*p* = .25) did not differ between ALI and ALN groups.
